# Preparations of Iron Dangerous in Certain Forms of Chlorosis

**Published:** 1844-04-20

**Authors:** 


*Preparations of Iron dangerous in certain forms of Chlorosis.* By M. Trousseau. (*Journal de Pharmacie*, June, 1843.) Iron has generally been regarded as a medicine which could not do harm in cases of chlorosis, but M. Trousseau's attention was powerfully attracted to the fact, that it is sometimes followed with most disastrous consequences, from meeting with two cases where the chlorotic affection not only resisted the preparations of iron, but where it seemed to light up a latent tubercular affection which rapidly carried off his patients. Since then he has remarked several cases; and, therefore, strongly recommends that iron be not administered in any case of chlorosis where tubercular affection has either been developed, or where there is a disposition to scrofula. As a general rule he states, that, if chlorosis occurs in a young girl about the age of puberty, in whom no scrofulous tumours have been observed since infancy or childhood, and who has never had hæmoptysis, iron, in large doses, may be safely administered with every prospect of soon removing the disease. If, however, there be any grounds for believing that there is a scrofulous tint, iron must be carefully abstained from, and the chlorotic affection must be endeavoured to be removed by change of air, tonic regimen, horse exercise, sulphurous preparations, &c. If the chlorotic affection has come on at the age of 25 to 35, there is reason to believe something seriously wrong, and preparations of iron are to be prescribed with caution; but if it has supervened suddenly on copious loss of blood, uterine hæmorrhage, or over-nursing, provided there be no scrofulous tendency, iron may be safely and freely prescribed.—*Edin. Med. and Surg. Jour.*

